# Melioidosis presenting as lymphadenitis: a case report

**DOI:** 10.1186/1756-0500-7-364

**Published:** 2014-06-14

**Authors:** Sanjeewa Wijekoon, Thushanthy Prasath, Enoka M Corea, Jayanthi P Elwitigala

**Affiliations:** 1 University Medical Unit, Colombo South Teaching Hospital, Kalubowila, Sri Lanka; 2 Department of Microbiology, Faculty of Medicine, University of Colombo, Colombo, Sri Lanka; 3 National TB Reference Laboratory, Chest Hospital, Welisara, Sri Lanka

**Keywords:** Melioidosis, Lymphadenitis, *Burkholderia pseudomallei*, Sri Lanka

## Abstract

**Background:**

Melioidosis is an infection caused by the facultative intracellular gram-negative bacterium; *Burkholderia pseudomallei*. It gives rise to protean clinical manifestations and has a varied prognosis. Although it was rare in Sri Lanka increasing numbers of cases are being reported with high morbidity and mortality. Here we report a case of melioidosis presenting with lymphadenitis which was diagnosed early and treated promptly with a good outcome.

**Case presentation:**

A 53-year-old Sinhalese woman with diabetes presented with fever and left sided painful inguinal lymphadenitis for one month. She had undergone incision and drainage of a thigh abscess three months previously and had been treated with a short course of antibiotics. There was no record that abscess material was tested microbiologically.

She had neutrophil leukocytosis and elevated inflammatory markers. Initial pus culture revealed a scanty growth of “Pseudomonas sp.” and *Escherichia coli* which were sensitive to ceftazidime and resistant to gentamicin.

Due to the history of diabetes, recurrent abscess formation and the suggestive sensitivity pattern of the bacterial isolates, we actively investigated for melioidosis. The bacterial isolate was subsequently identified as *B. pseudomallei* by polymerase chain reaction and antibodies to melioidin antigen were found to be raised at a titre of 1:160.

The patient was treated with high dose intravenous ceftazidime for four weeks followed by eradication therapy with cotrimoxazole and doxycycline. As the patient was intolerant to cotrimoxazole, the antibiotics were changed to a combination of co-amoxyclav and doxycycline and continued for 12 weeks. The patient was well after 6 months without any relapse.

**Conclusions:**

Melioidosis is an emerging infection in South Asia. It may present with recurrent abscesses. Therefore it is very important to send pus for culture whenever an abscess is drained. However, it should be noted that the reporting laboratory may be unfamiliar with this bacterium and the isolate may be misidentified as *Pseudomonas* or even *E. coli*. Melioidosis should be suspected when an isolate with the typical antibiotic sensitivity pattern of ceftazidime sensitivity and gentamicin resistance is cultured, especially in a patient with diabetes. This will expedite diagnosis and prompt treatment leading to an excellent prognosis.

## Background

Melioidosis is an infection caused by the facultative intracellular gram-negative bacterium; *Burkholderia pseudomallei*. Clinical features of melioidosis are highly variable. They range from asymptomatic disease, localized skin ulcers or abscesses and acute fulminant septicaemia to chronic infection. Predisposing factors for melioidosis include chronic diseases such as diabetes, chronic renal failure, chronic lung disease and alcoholism [1-3]. Infection spreads by percutaneous inoculation, inhalation, or ingestion. Melioidosis is endemic in tropical and subtropical zones of South East Asia and Northern Australia [1,2]. Although it is rare in Sri Lanka, increasing numbers of cases are being reported [4-10] and many of them were fatal [4-6,9]. Timely diagnosis and prompt institution of correct antibiotics will prevent high morbidity and mortality. Here we report a case of melioidosis, presenting as lymphadenitis, which was diagnosed reasonably early and treated aggressively with a very good prognosis.

## Case presentation

A 53-year-old Sinhalese woman from Chilaw in the North Western Province of Sri Lanka, presented with intermittent high fever with chills for one month and a painful left inguinal mass for two weeks. She had diabetes.

She had undergone an incision and drainage of a left deep thigh abscess three months previously and had been treated with a short course of antibiotics. There was no record that abscess material was tested microbiologically.

On examination, she was febrile with the temperature of 103°F. She had a tender, fluctuant mass in the left inguinal region. The rest of the examination was unremarkable.

There was a raised erythrocyte sedimentation rate (ESR) of 80 mm in the first hour and a very high C-reactive protein (CRP) level of 208 mg/dl. The white cell count was 19 × 10^9^/L with 75% neutrophils. Liver function and renal function were normal. Ultrasound scan revealed a left inguinal abscess. Excision biopsy of the abscess was done and samples were sent for histology, bacterial culture and antibiotic sensitivity testing, microscopy for acid fast bacilli and culture for tuberculosis.

Histology revealed chronic lymphadenitis with perinodal abscess formation. There were no acid fast bacilli in the direct smear. Initial culture results showed a scanty growth of *Pseudomonas* sp. and *E. coli* which was sensitive to ceftazidime and treatment commenced according to the sensitivity pattern.

However, since the *Pseudomonas* isolate was resistant to gentamicin and the patient had a history of recurrent abscess formation, melioidosis was considered in the differential diagnosis and the bacterial isolates were sent to the Department of Microbiology, Faculty of Medicine, University of Colombo for identification.

Bacterial colonies on blood agar were pin point in size after overnight incubation developing into 1-2 mm, white, umbonate colonies after 48 hours. On MacConkey agar the isolate was salmon pink, which may account for the erroneous conclusion that the “Pseudomonas” isolate was mixed with *E.coli*. The colonies had the characteristic musty, earthy odour of *B. pseudomallei* and were slowly oxidase positive*.* Gram stain appearance revealed the typical safety pin appearance. The isolate was resistant to gentamicin, polymyxin and colisitin and sensitive to co-amoxyclav. Subcultures of the isolate were couriered to a reference laboratory where they were confirmed as *B. pseudomallei* by polymerase chain reaction (PCR). Serum antibodies to melioidin antigen using an in-house indirect haemagglutination (IHA) test based on that described by Alexander *et al.* with antigen prepared from local strains of *B.pseudomallei*[11] were positive at a titre of 1:160.

After confirmation of the diagnosis of melioidosis, it was decided to treat her with intravenous ceftazidime for four weeks. There was excellent clinical improvement with normalization of markers of inflammation. Eradication therapy with oral co-trimoxazole and doxycycline was started on the third week, overlapping with the intravenous ceftazidime for one week. The patient tolerated the medications well.

The patient was discharged after completion of four weeks of intravenous ceftazidime with a plan to continue oral antibiotics for 12 weeks. However she presented to the outpatient follow up 2 weeks after discharge with an itchy rash which was presumed to be an adverse effect of co-trimoxazole. The rash improved after co-trimoxazole was replaced by co-amoxyclav. The patient was able to tolerate the co-amoxyclav/doxycycline combination and completed 12 weeks of eradication therapy. At the time of this writing she was well without evidence of recurrence or relapse.

## Conclusions

*B. pseudomallei* infections are known for their protean manifestations, ranging from septicaemia and pneumonia to asymptomatic infections, localised ulcers or abscesses. Diabetes has been found to be the single most common predisposing factor for melioidosis [2,3]. The disease occurs mainly in rural areas and is associated with occupational exposure to soil and surface water. In the majority of cases it probably goes undiagnosed and untreated.

Our patient had diabetes but she did not have any known exposure to soil or water. There was no history of travelling to a known endemic area suggesting that she had acquired the disease in Sri Lanka and that the disease is more prevalent than previously thought.

Ten cases [4-10] of melioidosis have been reported in Sri Lanka; the first, in an English tea broker in 1927. Most of them were fatal with only 4 survivors [7-10]. Delay in diagnosis may have been a factor contributing to the high mortality.

In our patient, we actively suspected melioidosis on the 4^th^ day after admission, when pus culture revealed a “pseudomonas species” resistant to gentamicin. The fact that she was still unwell, three months after surgery and antibiotics for a deep thigh abscess, strengthened our suspicion. Rapid initiation of appropriate therapy would have contributed to her good prognosis.

Isolation of *B. pseudomallei* in culture is essential to confirm the initial clinical diagnosis. Our patient may have been diagnosed three months previously if aspirated pus had been sent for microbiological examination. It is also important to note that, if the reporting laboratory is unfamiliar with this bacterium, *B. pseudomallei* may be reported as “pseudomonas species”. Therefore, if melioidosis is suspected, further bacterial identification should be requested.

Our patient had only intravenous ceftazidime for four weeks in the acute phase. Combination of co-trimoxazole with ceftazidime is not recommended in the acute phase as there is no added benefit [12]. Oral co-trimoxazole and doxycycline was chosen for the eradication phase of antibiotics. They were overlapped with ceftazidime for one week as this is the vulnerable period where reactivation can occur [12].

This case illustrates the fact that a high degree of clinical suspicion is needed to diagnose melioidosis at an early stage. When culture of patient specimens yields a “pseudomonas species” which is resistant to gentamicin, melioidosis should be actively excluded, especially in a diabetic.

Use of a selective medium such as Ashdown’s agar and alerting the microbiological community to the characteristic microscopic and colony morphology and resistance pattern of *B.pseudomallei* may help to improve the detection rate of melioidosis in the country.

A long course of intravenous antibiotics in the acute phase, overlapped and followed by a prolonged course of a combination of oral antibiotics is needed to improve the prognosis of this potentially fatal, emerging infection.

## Consent

Written informed consent was obtained from the patient for publication of this Case Report and any accompanying images. A copy of the written consent is available for review by the Editor –in-Chief of this journal.

## Abbreviations

PCR: Polymerase chain reaction; WBC: White blood count; ESR: Erythrocyte sedimentation rate; CRP: C-reactive protein; IHA: Indirect haemagglutination test.

## Competing interests

The authors declare that they have no competing interests.

## Authors’ contributions

SW made the clinical diagnosis, managed the patient and supervised the manuscript drafting. TP drafted the manuscript, reviewed the literature and was involved in management of the patient. EMC carried out identification of the isolate and performed the IHA and supervised manuscript drafting. JPE carried out the initial microbiological diagnosis and was involved in the early phase of the management of the patient. All authors have read and approved the final manuscript.

## Authors’ information

SW – MBBS, MD, − Consultant Physician, Senior Lecturer in Medicine, University Medical Unit, Colombo South Teaching Hospital, Sri Lanka. TP – MBBS – Registrar in Medicine, University Medical Unit, Colombo South Teaching Hospital, Sri Lanka. EMC – MBBS, Dip (Med Micro), MD (Med Micro) – Senior Lecturer in Department of Microbiology, Faculty of Medicine, University of Colombo, Sri Lanka. JPE – MBBS, Dip (Med Micro), MD (Med Micro) – Consultant Microbiologist, National TB Reference Laboratory, Welisara, Sri Lanka.
